# Dataset for petroleum based stock markets and GAUSS codes for SAMEM

**DOI:** 10.1016/j.dib.2016.10.031

**Published:** 2016-11-30

**Authors:** Ahmed A.A. Khalifa, Pietro Bertuccelli, Edoardo Otranto

**Affiliations:** a College of Business and Economics, Qatar University, Doha, Qatar; b Università degli Studi di Messina, Italy; c University of Messina, Italy

**Keywords:** GAUSS codes, Petroleum based stock markets, Volatility, SAMEM, Resources economics

## Abstract

This article includes a unique data set of a balanced daily (Monday, Tuesday and Wednesday) for oil and natural gas volatility and the oil rich economies’ stock markets for Saudi Arabia, Qatar, Kuwait, Abu Dhabi, Dubai, Bahrain and Oman, using daily data over the period spanning Oct. 18, 2006–July 30, 2015. Additionally, we have included unique GAUSS codes for estimating the spillover asymmetric multiplicative error model (SAMEM) with application to Petroleum-Based Stock Market. The data, the model and the codes have many applications in business and social science.

**Specifications Table**

**Table:** 

Subject area	Estimating Asymmetric multiplicative error model using GAUSS with application of petroleum based markets
More specific subject area	GAUSS code for estimating Asymmetric multiplicative error model
Type of data	Table, Excel file, text file
How data was acquired	Bloomberg data
Data format	Raw, filtered, analyzed, estimated
Experimental factors	Balanced series
Experimental features	Secondary data for the selected factors and markets
Data source location	are available with this article in the supplementary file (zip file)
Data accessibility	Data are available with this article in the supplementary file (zip file)

**Value of the data**

• The data can be used for investors, portfolio managers and for the researchers in economics and finance fields.
• Unique GAUSS codes of estimating Spillover Asymmetric Multiplicative Error Model that have many applications in finance, economics and social science.
• Through the GAUSS code, the researcher can test the interactions across all financial markets with and without crisis.

## 1 Data

The data set includes a balanced daily (Monday, Tuesday and Wednesday) for oil and natural gas volatility and the oil rich economies’ stock markets for Saudi Arabia, Qatar, Kuwait, Abu Dhabi, Dubai, Bahrain and Oman, using daily data over the period spanning Oct. 18, 2006–July 30, 2015 (a total of 3055 observations). The GCC indices under consideration are the Saudi Arabian Tadawul All Share Index (Saudi Arabia-TASI, hereafter), the Kuwait Stock Exchange Index, (Kuwait-SE), the Dubai General Index (Dubai-DFM), the Abu Dhabi General Index (Abu Dhabi-ADX), the Qatar Doha Securities Market (Qatar-QD), the Oman MSM30 Index (Oman-MSM30), Bahrain stock index (Bahrain-BE), the oil price (WTI-Oil) and the natural gas price (NG). a summary of the selected series is in Appendix A, Table 1. Additionally, we have included unique GAUSS codes for estimating the SAMEM. The data, the model and the codes have many applications in business and social science. The formula used to estimate volatility, Parkinson [6], is (ln(Max)−ln(Min))*(1/4*ln(2))^0.5.^1^ The choice of the sample period is motivated by the desire to capture the interactions among the markets over the years that are mostly characterized by several crisis and number of disruptions in the selected markets. With respect to the GAUSS codes, they are written and are tested to overcome the shortage in estimating the SAMEM model, for more information about the basic model of MEM, see Engle and Gallo [2]. Selected results of the estimated model is included in Appendix A (Table 2).

## 2 Experimental design, materials and methods

The data set is collected from a secondary source the period spanning Oct. 18, 2006–July 30, 2015 (a total of 3055 observations). The is collected from Bloomberg. It means, it is not experimental data. With respect to the SAMEM codes, they are written in GAUSS for the first time and they are tested and applied for spillover of volatility across the petroleum-based stock markets. The SEMEM model can be summarized as follows;

Both of the data set and the codes are provided in the supplementary documents.

With respect to the code of the SAMEM model, see Otranto [1,^3^–5], it is written based on the In the basic MEM model, the volatility $y_{t}$ of a certain financial time series is modeled as the product of a time varying scale factor $\mu_{t}$, which represents the conditional mean of $y_{t}$, which follows a GARCH-type dynamic, and a positive valued error $\epsilon_{t}$:

(1) $$y_{\mathrm{it}}=\;\mu_{\mathrm{it}}\epsilon_{t},\;\epsilon_{t}|\psi_{t-1}\sim \mathrm{Ga}\mathrm{mma}\left(a,\frac{1}{a}\right)\mathrm{for}\;\mathrm{each}\;t$$

(2) $$\mu_{\mathrm{it}}=\xi_{t}+\sum_{i=1}^{n}\xi_{i,t}$$

(3) $$\xi_{t}=\omega +\sum_{h=1}^{p_{i}}\alpha_{0,h}y_{t-h}+\;\sum_{j=1}^{q_{i}}\beta_{0,j}\xi_{t-j}+\gamma_{0}D_{0,t-1}y_{t-1}$$

(4) $$\xi_{i,t}=\;\sum_{h=1}^{p_{i}}\alpha_{i,h}x_{i,t-h}+\;\sum_{j=1}^{q_{i}}\beta_{i,j}\xi_{i,t-j}+\gamma_{j}D_{i,t-1}x_{i,t-1}$$

$$D_{t}=\left\{\begin{array}{cc} 1 & if\;r_{t}<0\\ 0 & if\;r_{t}>0 \end{array}\right\}$$

Following Eqs. (1), (2), (3), (4), the basic version of the multiplicative error model (MEM) has been developed to upgrade the GARCH (p,q)model to include the effects of the volatility of other markets in each equation of volatility as follows:

(5) $$\mu_{\mathrm{it}}=\omega +\sum_{h=1}^{p_{i}}\alpha_{0,h}y_{t-h}+\;\sum_{j=1}^{q_{i}}\beta_{0,j}\xi_{t-j}+\gamma_{0}D_{0,t-1}y_{t-1}+\sum_{h=1}^{p_{i}}\alpha_{i,h}x_{i,t-h}+\;\sum_{j=1}^{q_{i}}\beta_{i,j}\xi_{i,t-j}+\gamma_{j}D_{i,t-1}x_{i,t-1}$$

Within this context, we estimate several models that include the dummy variables during the crises and instability periods. For example, we have included a dummy variable for the global financial crisis (or other events such as the European debt crisis, the geopolitical instability in the Middle East and in the Arabian Gulf region).^2^ With respect to the asymmetric effects, this study will include two variables of the interactions between the volatility and dummies for negative returns ($D_{r,t-1<0}$) and positive returns days $D_{r,t-1>0}$ and the interaction between $D_{t-1}$ and asymmetric effects. For more details about the model see [4].

## Figures and Tables

**Table 1:** Descriptive statistics for the selected markets.

	VOL_Oil	VOL_NG	VOL_SA	VOL_KUW	VOL_QA	VOL_AD	VOL_DUB	VOL_OM	VOL_BAH
Mean	0.007	0.009	0.003	0.002	0.002	0.002	0.004	0.002	0.001
Median	0.005	0.008	0.002	0.001	0.002	0.002	0.003	0.001	0.001
Maximum	0.048	0.040	0.022	0.008	0.020	0.018	0.026	0.018	0.013
Minimum	0.001	0.002	0.000	0.000	0.000	0.000	0.001	0.000	0.000
Skewness	2.587	1.868	2.893	2.222	3.123	2.928	2.604	3.907	3.116
Kurtosis	13.851	9.190	13.950	9.120	16.587	15.994	12.588	24.539	24.599
Jarque-Bera	18397	6655	19524	7281	28464	25857	15156	66830	64325
Obs.	3055	3055	3055	3055	3055	3055	3055	3055	3055

*Notes*: VOL; Volatility, NG; Natural gas, S; Saudi Arabia, KU; Kuwait, AD; Abu Dhabi, D; Dubai, QA; Qatar, OM; Oman, BA, Bahrain.

**Table 2:** The Spillover Multiplicative Error Model results selected pairs of selected petroleum and petroleum-based stock markets and energy products (Kuwait-Natural Gas, Dubai-Natural Gas and Abu Dhabi-Natural Gas).

**Coefficients**	**KU-NG Without Exo.**	**KU-NG -Exo1**$\delta_{1}$	**KU-NG –Exo2**$\delta_{2}$	**DU-NG Without Exo.**	**DU-NG -Exo1**$\delta_{1}$	**DU-NG –Exo2**$\delta_{2}$	**AD-NG Without Exo.**	**AD-NG -Exo1**$\delta_{1}$	**AD-NG –Exo2**$\delta_{2}$
ω	0.138⁎	0.123	0.117	0.212	0.208	0.127	6.63	8.29	6.305
	(0.073)	(0.039)	(0.044)	(0.118)	(0.126)	(0.065)	(1.13)	(0.99)	(2.036)
$\alpha_{0}$	0.443	0.434	0.444	0.468	0.468	0.477	0.47	0.48	0.473
	(0.020)	(0.021)	(0.020)	(0.023)	(0.023)	(0.022)	(0.02)	(0.02)	(0.023)
$\beta_{0}$	0.432	0.428	0.428	0.428	0.424	0.398	0.41	0.39	0.387
	(0.029)	(0.029)	(0.027)	(0.031)	(0.032)	(0.031)	(0.03)	(0.03)	(0.033)
$\gamma_{0}$	0.000	0.006	0.000	0.000	0.000	0.000	0.02	0.02	0.022
	(0.000)	(0.006)	(0)	(0.000)	(0.000)	(0.000)	(0.01)	(0.01)	(0.007)
a	27.287	27.377	27.311	25.776	25.818	25.963	17.94	18.03	18.047
	(0.694)	(0.696)	(0.695)	(0.655)	(0.656)	(0.660)	(0.46)	(0.46)	(0.458)
$\alpha_{1}$	0.990	0.992	0.992	0.986	0.987	0.992	0.53	0.00	0.655
	(0.004)	(0.002)	(0.003)	(0.006)	(0.007)	(0.004)	(0.06)	(0.00)	(0.325)
$\beta_{1}$	0.001	0.001	0.001	0.002	0.002	0.001	0.01	0.00	0.008
	(0.000)	(0.000)	(0.000)	(0.001)	(0.001)	(0.000)	(0.01)	(0.00)	(0.004)
$\gamma_{1}$	0.000	0.000	0.000	0.000	0.000	0.000	0.00	0.01	0.003
	(0.00)	(0.00)	(0.00)	(0.000)	(0.000)	(0.00)	(0.00)	(0.00)	(0.004)
$\delta_{1}$**- GFC**		1.153			1.252			2.07	
		(0.385)			(0.573)			(0.58)	
$\delta_{2}-I\mathrm{ran}$			0.511			2.847			2.283
			(0.00)			(0.641)			(0.573)
**log- likelihood**	−11,838	−11,833	−11,837	−13,222	−13,220	−13,211	−12,742	−12,735	−12,733
**AIC**	7.75	7.75	7.75	8.66	8.66	8.65	8.34	8.34	8.34
**BIC**	7.77	7.77	7.77	8.67	8.68	8.67	8.36	8.36	8.36
**RMSE**	12.76	12.73	12.77	20.30	20.28	20.27	17.44	17.40	17.40
**MAE**	9.07	9.05	9.07	14.57	14.54	14.54	12.66	12.64	12.64

⁎ Means that the coefficients are significant at the less than 5% significance level.

## Appendix A

See Table 1, Table 2 here.

## Footnotes

1 For more details about this measure of volatility, visit Khalifa et al. [3,4].

2 As in footnote (1).
