# Supplementary material for: Dataset for petroleum based stock markets and GAUSS codes for SAMEM
Source: Data Brief. 2016 Nov 30;10:421–5. doi: 10.1016/j.dib.2016.10.031 (PMC5192250; doi:10.1016/j.dib.2016.10.031)
Supplement: Supplementary file 1 — Supplementary material [file mmc1.pdf]

AUTHOR DECLARATION TEMPLATE

We wish to draw the attention of the Editor to the following facts which may be considered as potential conflicts of interest and to significant financial contributions to this work. [OR] We wish to confirm that there are no known conflicts of interest associated with this publication and there has been no significant financial support for this work that could have influenced its outcome. We confirm that the manuscript has been read and approved by all named authors and that there are no other persons who satisfied the criteria for authorship but are not listed. We further confirm that the order of authors listed in the manuscript has been approved by all of us. We confirm that we have given due consideration to the protection of intellectual property associated with this work and that there are no impediments to publication, including the timing of publication, with respect to intellectual property. In so doing we confirm that we have followed the regulations of our institutions concerning intellectual property. We further confirm that any aspect of the work covered in this manuscript that has involved either experimental animals or human patients has been conducted with the ethical approval of all relevant bodies and that such approvals are acknowledged within the manuscript

We understand that the Corresponding Author is the sole contact for the Editorial process (including Editorial Manager and direct communications with the office). He/she is responsible for communicating with the other authors about progress, submissions of revisions and final approval of proofs. We confirm that we have provided a current, correct email address which is accessible by the Corresponding Author and which has been configured to accept email from

Signed by all authors as follows:

Authors: Ahmed A.A. Khalifa (1),

Pietro Bertuccelli (2),

Edoardo Otranto (3)

Affiliations:

1 College of Business and Economics, Qatar University, Doha, Qatar, Email: aliabdelkh@qu.edu.qa, Tel: +974-74015848, Fax: +974-4403-5081

2 Università degli Studi di Messina, Email: pietro.bertuccelli@gmail.com

3 University of Messina , Italy, Email; eotranto@unime.it

FACTSHEET

Conflict of Interest

Transparency and objectivity are essential in scientific research and the peer review process.

When an investigator, author, editor, or reviewer has a financial/personal interest or belief that could affect his/her objectivity, or inappropriately influence his/her actions, a potential conflict of interest exists. Such relationships are also known as dual commitments, competing interests, or competing loyalties.[1,2]

The most obvious conflicts of interest are financial relationships such as:
- **Direct:** employment, stock ownership, grants, patents.
- **Indirect:** honoraria, consultancies to sponsoring organizations, mutual fund ownership, paid expert testimony.[2]

Undeclared financial conflicts may seriously undermine the credibility of the journal, the authors, and the science itself.[2] An example might be an investigator who owns stock in a pharmaceutical company that is commissioning the research.

Conflicts can also exist as a result of personal relationships, academic competition, and intellectual passion.[2] An example might be a researcher who has:
- A relative who works at the company whose product the researcher is evaluating.
- A self-serving stake in the research results (e.g. potential promotion/career advancement based on outcomes).
- Personal beliefs that are in direct conflict with the topic he/she is researching.

Not all relationships represent a true conflict of interest–conflicts can be potential or actual.[1,2] Some considerations that should be taken into account include: whether the person's association with the organization interferes with their ability to carry out the research or paper without bias; and whether the relationship, when later revealed, make a reasonable reader feel deceived or misled.[3]

Full disclosure about a relationship that could constitute a conflict–even if the person doesn't believe it affects their judgment–should be reported to the institution's ethics group and to the journal editor to which a paper is submitted. All publishers require disclosure in the form of a cover letter and/or footnote in the manuscript.

A journal may use disclosures as a basis for editorial decisions and may publish them if they are believed to be important to readers in judging the manuscript. Likewise, the journal may decide not to publish on the basis of the declared conflict.

According to the U.S. Office of Research Integrity, having a conflict of interest is not in itself unethical, and there are some that are unavoidable.[1] Full transparency is always the best course of action, and, if in doubt, disclose.

Guide to Conflict of Interest and How to Prevent It*

Action	What is it?	Is it unethical?	What should you do?
**An undisclosed relationship that may pose a conflict of interest**	Neglecting to disclose a relationship with a person or organization that could affect one's objectivity, or inappropriately influence one's actions.	**Yes.**<br><br>Some relationships do not necessarily present a conflict. Participants in the peer-review and publication process must disclose relationships that could be viewed as potential conflicts of interest.[2]	■ When submitting a paper, state explicitly whether potential conflicts do or do not exist.<br>■ Indicate this in the manuscript on a conflict-of-interest notification page, with additional detail. If necessary, include a cover letter with the manuscript.<br>■ Investigators must disclose potential conflicts to study participants and should state in the manuscript whether they have done so.<br>■ Reviewers must also disclose any conflicts that could bias their opinions of the manuscript.[2]
**An undisclosed funding source that may pose a conflict of interest**	Neglecting to disclose the role of the study sponsor(s), if any, in study design; in the collection, analysis, and interpretation of data; in the writing of the report; and in the decision to submit the paper for publication.	**Yes.**<br><br>Undeclared financial conflicts may seriously undermine the credibility of the journal, the authors, and the science itself.[2]	■ When submitting a paper, a declaration (with the heading 'Role of the funding source') should be made in a separate section of the text and placed before the References.<br>■ Describe the role of the study sponsor(s), if any, in study design; in the collection, analysis, and interpretation of data; in the writing of the report; and in the decision to submit the paper for publication.<br>■ Editors may request that authors of a study funded by an agency with a proprietary or financial interest in the outcome sign a statement, such as "I had full access to all of the data in this study and I take complete responsibility for the integrity of the data and the accuracy of the data analysis."[2]

*When in doubt, always consult with your professor, advisor, or someone in a position of authority who can guide you to the right course of action.

References

1. Office of Research Integrity U.S. Department of Health and Human Services. A brief overview on Conflict of Interests. Available at: http://ori.hhs.gov/plagiarism-35. Accessed on September 3, 2012.
2. International Committee of Medical Journal Editors. Uniform Requirements for Manuscripts Submitted to Biomedical Journals: Ethical Considerations in the Conduct and Reporting of Research: Conflicts of Interest. Available at: http://www.icmje.org/ethical_4conflicts.html. Accessed on September 2, 2012.
3. Committee on Publication Ethics (COPE). Committee on Publication Ethics (COPE). Guidelines on Good Publication Practice. 1999. Available at: http://publicationethics.org/static/1999/1999pdf13.pdf. Accessed on September 6, 2012.
